# Pointillisme à la Signac and Construction of a Quantum Fiber Bundle Over Convex Bodies

**DOI:** 10.1007/s10701-023-00681-2

**Published:** 2023-03-29

**Authors:** Maurice de Gosson, Charlyne de Gosson

**Affiliations:** Faculty of Mathematics (NuHAG), University of Vienna, Oskar-Morgenstern-Platz 1, 1090 Vienna, Austria

**Keywords:** Lagrangian frame, Symplectic group, Polar duality, Gaussian wavepackets, Wigner transform, Quantum fiber bundle

## Abstract

We use the notion of polar duality from convex geometry and the theory of Lagrangian planes from symplectic geometry to construct a fiber bundle over ellipsoids that can be viewed as a quantum-mechanical substitute for the classical symplectic phase space. The total space of this fiber bundle consists of geometric quantum states, products of convex bodies carried by Lagrangian planes by their polar duals with respect to a second transversal Lagrangian plane. Using the theory of the John ellipsoid we relate these geometric quantum states to the notion of “quantum blobs” introduced in previous work; quantum blobs are the smallest symplectic invariant regions of the phase space compatible with the uncertainty principle. We show that the set of equivalence classes of unitarily related geometric quantum states is in a one-to-one correspondence with the set of all Gaussian wavepackets. We emphasize that the uncertainty principle appears in this paper as geometric property of the states we define, and is not expressed in terms of variances and covariances, the use of which was criticized by Hilgevoord and Uffink.

## Introduction

The Wigner transform and its variants (Bargmann transform, Gabor transform) have in common that they associate to a *function* on configuration space another function defined on phase space. For instance, in quantum mechanics the Wigner transform takes an arbitrary probability amplitude $\psi$ (the wavefunction) to a quasi-probability density on $W\psi$ phase space whose marginals give the true probabilities of finding the quantum system under consideration in both some localization of configuration space and a localization in momentum space. In the present work we suggest a novel “Configuration space <–> phase space correspondence” of a more general nature: instead of starting with a functions to which we associate quasi-probability densities, we start with subsets of *x*-space (usually convex bodies, or even ellipsoids) to which we make correspond subsets in phase space; these are obtained using the notion of polar dually from convex geometry. Our constructions are somewhat related to the so-called “Pauli reconstruction problem”, which can be formulated in the Wigner formalism as follows: knowing the probability densities $|\psi |^{2}$ and $|\widehat{\psi }|^{2}$ ($\widehat{\psi }$ the Fourier transform of $\psi$) can we determine *uniquely* $\psi$? (It is known that the answer to this question is generally negative). As we will see, our construction will give a geometric setting to this question and the involved difficulties and ambiguities. What is remarkable and new in our treatment is that we do not assume that the “quantum states” we define by this procedure are associated in any way (as is the case in standard quantum mechanics) to any notion of wavefunction. This is where our approach departs from the usual one. One has first to understand that as opposed to a widespread belief, Quantum Mechanics does not say that particles are waves. While the wave interpretation was de Broglie’s original point of view, it is today is untenable. Of course particle dynamics may be described using waves as solutions of Schrödinger’s equation, but this is not the same as saying particles *are* waves! Quantum mechanics is a probabilistic theory from which we can extract consequences on the statistical behavior of many measurements performed on a great number of equally prepared systems. The real issue is the calculation of probabilities, and the objects used to study them (variances, spreading, covariances, mean values...) The uncertainty inequalities are statements about the variances and covariances of the random variables corresponding to independently measured position and momentum in an ensemble of equally prepared particles. We emphasize that they are neither a statement about the measure of both momentum and position of a single particle nor an effect of the interaction with a measurement device as early interpretations of the Heisenberg inequalities seemed to suggest (Uffink and Hilgevoord have shown [19, 20] that the use variances and covariances to describe the statistical properties of a quantum system is valid only for Gaussian or almost Gaussian states).

### Pointillisme à la Signac and Phase Space Pixels

In two brilliant publications [8, 9] Jeremy Butterfield dismisses what he calls *pointillisme, *that is the view that mathematical *points* make sense in physics. We totally agree with Butterfield’s views and assume in this paper that the basic elements of configuration space (i.e. physical space, and its multi-dimensional extensions) are infinitesimal regions with non-zero volume. Indeed, in practice we can never experimentally determine a point in physical space with absolute precision; as Gazeau [18] humorously notes

Nothing is mathematically exact from the phsical point of view.

In fact the notion of point-like particle is a mathematical abstraction, which we can (in principle) approximate with arbitrary accuracy. However, these regions cannot be made arbitrarily small, because the uncertainty principle would then lead to violations of special relativity (at least for massive particles) since in the limit $\Delta x\rightarrow 0$ the Heisenberg relation $\Delta p\Delta x\sim \hbar$ leads to values of $\Delta p$ exceeding the speed of light. Our view in a sense restores pointillisme as meant by the neo-impressionist painter Paul Signac, who used small, distinct dots of color which he applied in patterns to form an image. We will show that this coarse graining of the usual configuration space leads, using an extended version of the geometric notion of polar duality, to a fiber bundle which can be viewed as a substitute for a quantum phase space. Admittedly, the term “quantum phase space” is usually perceived as a heresy in the physics community: there can’t be any phase space in quantum mechanics since the notion of a well-defined point does not make sense because of the uncertainty principle. Dirac himself dismissed in 1945 in a letter to Moyal (in [22]), even the suggestion that quantum mechanics can be expressed in terms of classical-valued phase space variables. Of course, as we know, Dirac was wrong, since the Wigner–Moyal–Weyl formalism, which deals with functions and operators defined on classical phase space, is one of the most powerful tools for expressing the laws of quantum mechanics. Still, the concept of *quantum phase space* itself is ambiguous, to say the least; the aim of this paper is to propose a substitute, which is a collection of fiber bundles. The simplest of these is the “canonical bundle”

1 $$\begin{aligned} \pi _{\text{can}}:{\text {Quant}}(n)\longrightarrow {\text {Conv}} (n) \end{aligned}$$

where ${\text {Conv}}(n)$ is the set of convex bodies in configuration space ${\mathbb {R}}_{x}^{n}$; the fiber over $X\in {\text {Conv}}(n)$ consists of the Cartesian products $X\times X^{\hbar }(x_{0})$ where $X^{\hbar }(p_{0})$ is the polar dual of *X* centered at $p_{0}\in {\mathbb {R}}_{p}^{n}$. For instance

$$\begin{aligned} \pi ^{-1}(B_{X}^{n}(x_{0}\sqrt{\hbar }))=\left\{ B_{X}^{n}(x_{0}\sqrt{\hbar })\times B_{P}^{n}(p_{0},\sqrt{\hbar }):p_{0}\in {\mathbb {R}}_{p}^{n}\right\} \end{aligned}$$

where $B_{X}^{n}(x_{0},\sqrt{\hbar })$ and $B_{P}^{n}(p_{0},\sqrt{\hbar })$ are balls with radius $\sqrt{\hbar }$ centered at $x_{0}$ and $p_{0}$; this reduces, in the limit $\hbar \rightarrow 0$, to the products $\{x_{0} \}\times {\mathbb {R}}_{p}^{n}$. We will draw several consequences from these definitions. In particular we will see that if we restrict the base space of the fiber bundle (1) to ellipsoids, then we have a continuous action of the unitary group $U(n,{\mathbb {C}})$ on ${\text {Quant}}(n)$ and that the homogeneous space ${\text {Quant}}(n)/U(n,{\mathbb {C}})$ can be identified with the set ${\text {Gauss}}(n)$ of all generalized Gaussian wavepackets on ${\mathbb {R}}_{x}^{n}$.
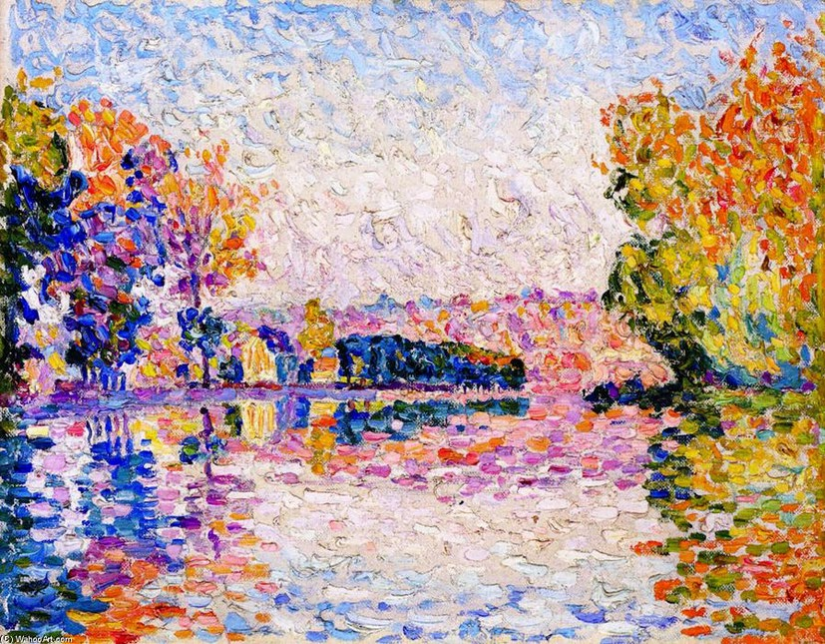


### Description of the Method: Heuristics

The aim of the present paper is to study, for an arbitrary number *n* of degrees of freedom, the properties of such “quantum state” and to relate them to the theory of Gaussian wavepackets; our study will unveil unexpected and beautiful geometric properties of quantum mechanics.

### Toolbox and Terminology

We introduced in [15] the geometric notion of Lagrangian polar duality in connection with the uncertainty principle of quantum mechanics; in a recent paper [16] we have detailed this results and given a rigorous mathematical study of this notion. As pointed out in [15] the underlying idea is that a quantum system localized in the position representation in a set *X* cannot be localized in the momentum representation in a set smaller than its polar dual $X^{\hbar }$; this is a geometric form of the uncertainty principle, independent of the notion of variance or covariance. Let us explain this a little bit more in detail. We live in a three-dimensional world where the state of a classical particle is described by its position vector (*x*, *y*, *z*) and by the vector of conjugate momenta $(p_{x},p_{y},p_{z})$, both at a given time *t*. This extends to many particle systems by introducing the generalized position and momentum vectors $x=(x_{1},\ldots ,x_{n})$ and $p=(p_{1},\ldots ,p_{n})$, and the phase space of that system is by definition the space ${\mathbb {R}}_{x}^{n}\times {\mathbb {R}}_{p} ^{n}\equiv {\mathbb {R}}^{2n}$ of all (*x*, *p*). This way of writing things explicitly singles out the two subspaces $\ell _{X}={\mathbb {R}}_{x}^{n}\times 0$ and $\ell _{P}=0\times {\mathbb {R}}_{p}^{n}$; however, as is already clear in classical (Hamiltonian) mechanics this “canonical” choice of frame $(\ell _{X},\ell _{P})$ has no reason to be privileged, and one can choose any other coordinate spaces to work with as long as these are obtained by symplectic transformations from the frame $(\ell _{X},\ell _{P})$. Such transformations will not take $\ell _{X}$ and $\ell _{P}$ to arbitrary *n*-dimensional linear subspaces of ${\mathbb {R}}^{2n}$, but rather to *Lagrangian planes* which have the property that the canonical symplectic form on ${\mathbb {R}}^{2n}$ vanishes identically on them. These subspaces play a central role in classical mechanics (they are the tangent spaces of the invariant tori of the integrable Hamiltonian systems [1]). Consider now a convex compact set $X_{\ell }$ with non-empty interior (for instance an ellipsoid) carried by a Lagrangian plane $\ell$. if, for instance, $\ell =\ell _{X}$ this convex body $X_{\ell }$ can be physically interpreted as a cloud of points in configuration space corresponding to a sequence of measurements. Assuming, for simplicity, that $X_{\ell }$ is centered at the origin, we next choose a second arbitrary Lagrangian plane $\ell ^{\prime }$ transversal to $\ell$ and define the polar dual $X_{\ell ^{\prime }}^{\hbar }$of $X_{\ell }$ with respect to $\ell ^{\prime }$ as being the set of all phase space points $z^{\prime }=(x,p^{\prime })$ such that $\omega (z,z^{\prime })\le \hbar$ for every $z=(x,p)$ in $X_{\ell }$. An elementary argument shows that $X_{\ell ^{\prime }}^{\hbar }$ is also a convex set (and in particular an ellipsoid if $X_{\ell }$ is). We will call the subset $X_{\ell }\times X_{\ell ^{\prime }}^{\hbar }$ of ${\mathbb {R}}^{2n}$ a *pure quantum state*. Admittedly, this definition of a quantum state is rather abstract. The reason will become clear to the reader in the course of this article, but there is a rather immediate (although hidden) motivation. It turns out that the Cartesian product $X_{\ell }\times X_{\ell ^{\prime }}^{\hbar }$ is always a convex set (because $X_{\ell }$ and $X_{\ell ^{\prime }}^{\hbar }$ are convex). As such it contains a unique maximum volume ellipsoid $\Omega$ (the “John ellipsoid”), and this ellipsoid is what we have called elsewhere [13, 17] a *quantum blob*, that is the image of a phase space ball with radius $\sqrt{\hbar }$ by a symplectic transformation. As we have shown in [12, 17] these quantum blobs represent the smallest phase space units compatible with the uncertainty (or indeterminacy) principle of quantum mechanics. In particular, a quantum blob can always (via the theory of the Wigner transform) be viewed as the covariance ellipsoid of a generalized Gaussian state.

Here is a basic example. Suppose that the configuration space is the *x* axis, in which case the classical phase space is just the *x*, *p* plane. The pseudo quantum phase space consists of parallelograms $X_{\ell }\times X_{\ell } ^{\hbar }$ where $\ell$ and $\ell ^{\prime }$ are two lines in the the *x*, *p* plane, $X_{\ell }$ is an interval in $\ell$ and $X_{\ell }^{\hbar }$ is the polar dual of $X_{\ell }$ with respect to $\ell ^{\prime }$. The latter is the set of points $z^{\prime }$ on $\ell ^{\prime }$ such that

$$\begin{aligned} \omega (z^{\prime },z)=- \begin{vmatrix} x^{\prime }&\quad x\\ p^{\prime }&\quad p \end{vmatrix} \le \hbar \end{aligned}$$

for all $z=(x,p)$ on $\ell$. If $\ell$ is the *x*-axis and $\ell ^{\prime }$ the *p*-axis this condition becomes $p^{\prime }x\le \hbar$ so $X_{\ell }^{\hbar }$ is the usual polar dual from convex geometry [15]. Choosing $X_{\ell _{X}}=[-\sqrt{\hbar /a},\sqrt{\hbar /a}]$ for some $a>0$ we have $X_{\ell _{P}}^{\hbar }=[-\sqrt{a\hbar },\sqrt{a\hbar }]$ so that $X_{\ell _{X} }\times X_{\ell _{P}}^{\hbar }$ is a parallelogram with area $4\hbar$ centered at the origin. Now, the largest ellipse contained in that parallelogram is the one with axes $X_{\ell _{X}}$ and $X_{\ell _{P}}^{\hbar }$ and thus has area $\pi \hbar$. To such an ellipse corresponds (via the theory of the Wigner transform) a unique (normalized) Gaussian wavepacket, namely

$$\begin{aligned} \psi (x)=\left( \tfrac{a}{\pi \hbar }\right) ^{1/4}e^{-ax^{2}/\hbar } \end{aligned}$$

which is a minimum uncertainty wavepacket. To our “quantum state” $X_{\ell _{X}}\times X_{\ell _{P}}^{\hbar }$ thus corresponds a basic object from quantum mechanics (a Gaussian wavepacket), but is a more general object than just this wavepacket.

#### Organization of the Paper

In Sect. 2 we review the basics of symplectic geometry we are using in this work; this includes a short discussion of the symplectic group and of the Lagrangian Grassmannian of the standard symplectic space. The main result is the transitivity of the action of the symplectic group on the pairs of transverse Lagrangian planes (Proposition 3). In Sect. 3 we begin with some preparatory geometric work, defining and studying the notion of Lagrangian polarity which is central to this work. Thereafter, in 4, we carefully define the notion of Lagrangian quantum state using our notion of polar duality. The main result is Proposition 13 where we describe the action of the symplectic group on the set of Lagrangian quantum states. In Sect. 5 we relate the notion of Lagrangian quantum state to the usual Gaussian wavepackets and their Wigner transforms; he main results are there Propositions 16 and 17 about Gaussian Lagrangian quantum states and the notion of quantum blob we introduced in previous work. Finally, we discuss in Sect. 6 perspectives for generalizing our results to the non-elliptic (= non-Gaussian) case, which would open the way for a total geometric description of general quantum states.

#### Notation

The configuration space of a system with *n* degrees of freedom will in general be written $\ell _{X}={\mathbb {R}}_{x}^{n}$, and its dual (the momentum space) $\ell _{P}={\mathbb {R}}_{p}^{n}$. The position variables will be written $x=(x_{1},\ldots ,x_{n})$ and the momentum variables $p=(p_{1},\ldots ,p_{n})$. The classical phase space ${\mathbb {R}}_{x}^{n}\times {\mathbb {R}}_{p}^{n}$ is identified with ${\mathbb {R}}^{2n}$ equipped with the inner product $p\cdot x=p_{1}x_{1}+\cdots +p_{n}x_{n}$ and with the standard symplectic form $\omega$ defined by $\omega (z,z^{\prime })=p\cdot x^{\prime }-p^{\prime }\cdot x$ if $z=(x,p)$, $z^{\prime }=(x^{\prime },p^{\prime })$.

## Some Symplectic Geometry

### The Symplectic Group ${\text {Sp}}(n)$

The standard symplectic form $\omega$ on ${\mathbb {R}}_{z}^{2n}\equiv {\mathbb {R}}_{x}^{n}\times {\mathbb {R}}_{p}^{n}$ can be written in matrix form as

$$\begin{aligned} \omega (z,z^{\prime })=Jz\cdot z^{\prime }=(z^{\prime })^{T}Jz \end{aligned}$$

where *J* is the standard symplectic matrix:

$$\begin{aligned} J= \begin{pmatrix} 0_{n\times n} &{}\quad I_{n\times n}\\ -I_{n\times n} &{}\quad 0_{n\times n} \end{pmatrix}. \end{aligned}$$

The associated symplectic group ${\text {Sp}}(n)$ consists of all linear automorphisms *S* of ${\mathbb {R}}_{z}^{2n}$ preserving the symplectic form: $\omega (Sz,Sz^{\prime })=\omega (z,z^{\prime })$ for all vectors $z,z^{\prime }$. The symplectic automorphisms will be identified with their matrices in the canonical basis; with this convention $S\in {\text {Sp}}(n)$ if and only it satisfies one of the equivalent identities $S^{T}JS=J$ or $SJS^{T}=J$. These relations imply [11] that a real $2n\times 2n$ matrix written in the block form

2 $$\begin{aligned} S= \begin{pmatrix} A &{}\quad B\\ C &{}\quad D \end{pmatrix} \end{aligned}$$

is symplectic if and only if the $n\times n$ blocks *A*, *B*, *C*, *D* satisfy the sets of equivalent conditions

3 $$\begin{aligned} A^{T}C , B^{T}D \textit{ symmetric, and }A^{T}D-C^{T}B&=I_{n\times n}\end{aligned}$$

4 $$\begin{aligned} AB^{T} , CD^{T} \textit{ symmetric, and }AD^{T}-BC^{T}&=I_{n\times \times n}. \end{aligned}$$

It follows that the inverse of $S\in {\text {Sp}}(n)$ has the simple form

5 $$\begin{aligned} S^{-1}= \begin{pmatrix} D^{T} &{}\quad -B^{T}\\ -C^{T} &{}\quad A^{T} \end{pmatrix}. \end{aligned}$$

The affine (or inhomogeneous) symplectic group is the semi-direct product

6 $$\begin{aligned} {\text {ISp}}(n)={\text {Sp}}(n)\ltimes {\mathbb {R}}^{2n}; \end{aligned}$$

it consists of all products $ST(z_{0})=T(Sz_{0})S$ where $S\in {\text {Sp}}(n)$ and $T(z_{0})$ is the translation operator $z\longmapsto z+z_{0}$ in ${\mathbb {R}}^{2n}$.

Recall [11] that the metaplectic group ${\text {Mp}}(n)$ is the unitary representation on $L^{2}({\mathbb {R}}_{x}^{n})$ of the double cover of the symplectic group ${\text {Sp}}(n)$. It is generated by the unitary operators $\widehat{J}$, $\widehat{V}_{P}$, and $\widehat{M}_{L.m}$ defined in the table below, where we denote $\pi ^{{\text {Mp}}}$ the projection ${\text {Mp}}(n)\longrightarrow {\text {Sp}}(n).$

**Table:** 

$\widehat{J}\psi (x)=\left( \tfrac{1}{2\pi i\hbar }\right) ^{n/2}\int e^{-\frac{1}{\hbar }x\cdot x^{\prime }}\psi (x^{\prime })d^{n}x^{\prime }$	$\overset{\pi ^{{\text {Mp}}}}{\longrightarrow }$	$$J= \begin{pmatrix} 0 &{}\quad I\\ -I &{}\quad 0 \end{pmatrix}$$
$\widehat{V}_{P}\psi (x)=e^{-\frac{i}{2\hbar }Px\cdot x}\psi (x)$	$\overset{\pi ^{{\text {Mp}}}}{\longrightarrow }$	$$V_{P}= \begin{pmatrix} I &{}\quad 0\\ -P &{}\quad I \end{pmatrix}$$
$\widehat{M}_{L.m}\psi (x)=i^{m}\sqrt{|\det L|}\psi (Lx)$	$\overset{\pi ^{{\text {Mp}}}}{\longrightarrow }$	$$M_{L}= \begin{pmatrix} L^{-1} &{}\quad 0\\ 0 &{}\quad L^{T} \end{pmatrix}.$$

In the last line of this table the integer *m* is defined modulo 4 and corresponds to a choice of the argument of the determinant $\det L$, reflecting the fact that ${\text {Mp}}(n)$ is a double covering of ${\text {Sp}}(n)$. For a complete study of ${\text {Mp}}(n)$ and its properties we refer to [11]. The non-homogeneous analogue of ${\text {Mp}}(n)$ is denoted ${\text {IMp}}(n)$; it consists of all operators $\widehat{S}\widehat{T}(z_{0})=\widehat{T}(Sz_{0})\widehat{S}$ where $\widehat{S}\in {\text {Mp}}(n)$, $z_{0}\in {\mathbb {R}}^{2n}$, and $\widehat{T}(z_{0})$ is the Heisenberg displacement operator:

$$\begin{aligned} \widehat{T}(x_{0},p_{0})\psi (x)=e^{\frac{i}{\hbar }(p_{0}\cdot x-\frac{1}{2}p_{0}\cdot x_{0})}\psi (x-x_{0}). \end{aligned}$$

The natural projection ${\text {IMp}}(n)\longrightarrow {\text {ISp}}(n)$ is defined by $\widehat{S}\widehat{T}(z_{0} )\longmapsto ST(z_{0})$.

### Lagrangian Planes and Frames

When $n=1$ the symplectic form is, up to the sign, the determinant function: $\omega (z,z^{\prime })=-\det (z,z^{\prime })$. It follows that $\omega (z,z^{\prime })=0$ when *z* and $z^{\prime }$ are colinear: the symplectic form vanishes along all lines through the origin. The notion of Lagrangian plane generalizes this property to arbitrary dimension *n*: a linear subspace $\ell$ of ${\mathbb {R}}^{2n}$ equipped with its symplectic form $\omega$ is called a *Lagrangian plane* if $\dim \ell =n$ and $\omega (z,z^{\prime })=0$ for all $z,z^{\prime }\in \ell$.

The most typical (but not most general) example of Lagrangian planes is given by the “coordinate Lagrangian planes”. They are obtained by picking out in the 2*n*-vector $z=(x_{1},\ldots ,x_{n};p_{1},\ldots ,p_{n})$ exactly *n* non-conjugate coordinates. For instance the set of all $(x_{1},\ldots ,x_{k},p_{k+1},\ldots ,p_{n})$ for $k<n$ are the coordinates of a Lagrangian plane in ${\mathbb {R}}^{2n}$.

The subspaces consisting of all $z=(x,p)$ such that $p=Ax$ for some symmetric matrix *A* is a Lagrangian plane: it has dimension *n* and

$$\begin{aligned} \omega (x,Ax;x^{\prime },Ax^{\prime })=Ax\cdot x^{\prime }-Ax^{\prime }\cdot x=0 \end{aligned}$$

since *A* is symmetric. More generally, a subspace $\ell$ of ${\mathbb {R}}^{2n}$ is a Lagrangian plane if and only we have

$$\begin{aligned} (x,p)\in \ell \text { if and only }Ax+Bp=0. \end{aligned}$$

where *A* and *B* are real $n\times n$ matrices satisfying one of the following sets of equivalent conditions

$$\begin{aligned} A^{T}B&=B^{T}A \textit{ and } A^{T}A+B^{T}B=I_{n\times n}\\ AB^{T}&=BA^{T} \textit{ and } AA^{T}+BB^{T}=I_{n\times n}. \end{aligned}$$

The set of all Lagrangian planes in the symplectic space $({\mathbb {R}} ^{2n},\omega )$ is called the *Lagrangian Grassmannian* and is denoted by ${\text {Lag}}(n)$.

#### Remark 1

There is an alternative way of interpreting Lagrangian planes as subspaces of ${\mathbb {C}}^{2n}$ on which the inner product $(z,z^{\prime })\longmapsto z\cdot (z^{\prime })^{*}$is real. In fact, the symplectic product $\omega (z,z^{\prime })$ can be written as $\omega (z,z^{\prime } )={\text {Im}}(z\cdot (z^{\prime })^{*})$ when $z=(x,p)$ and $z^{\prime }=(x^{\prime },p^{\prime })$ are identified with the complex vectors $x+ip$ and $x^{\prime }+ip^{\prime }$ in ${\mathbb {C}}^{n}$. Lagrangian planes then correspond to the *n*-dimensional subspaces for which $z\cdot (z^{\prime })^{*}$ is a real number.

In the phase plane ${\mathbb {R}}^{2}$ every line through the origin can be taken to any other such line using a rotation. There is a similar property in arbitrary dimension *n*. A symplectic automorphism *U* is called a *symplectic* *rotation* if $U\in {\text {Sp}}(n)\cap O(2n,{\mathbb {R}})$ where $O(2n,{\mathbb {R}})$ is the usual orthogonal group. In the case $n=1$ this is just the usual rotation group $SO(2n,{\mathbb {R}})$. We denote by *U*(*n*) the group of all symplectic rotations; one shows [11] that *U*(*n*) is the image in ${\text {Sp}}(n)$ of the complex unitary group $U(n,{\mathbb {C}})$ by the embedding

$$\begin{aligned} \iota :A+iB\longmapsto \begin{pmatrix} A &{}\quad B\\ -B &{}\quad A \end{pmatrix}. \end{aligned}$$

A matrix $$\begin{pmatrix} A &{}\quad B\\ -B &{}\quad A \end{pmatrix}$$ is thus a symplectic rotation if and only if the blocks *A* and *B* satisfy the conditions

7 $$\begin{aligned} A^{T}B&=B^{T}A\text { and }A^{T}A+B^{T}B=I \end{aligned}$$

8 $$\begin{aligned} AB^{T}&=BA^{T}\text { and }AA^{T}+BB^{T}=I \end{aligned}$$

in view of (3), (4).

Let $\ell$ be a Lagrangian plane in $({\mathbb {R}}^{2n},\omega )$: $\ell \in {\text {Lag}}(n)$. For every symplectic transformation $S\in {\text {Sp}}(n)$ the image $S\ell$ is also a Lagrangian plane: we clearly have $\dim S\ell =n$ and $\omega (Sz,Sz^{\prime })=\omega (z,z^{\prime })=0$ for all $z,z^{\prime }\in \ell$. We thus have a natural group action

9 $$\begin{aligned} {\text {Sp}}(n)\times {\text {Lag}}(n)\longrightarrow {\text {Lag}}(n) \end{aligned}$$

which induces, by restriction, an action

10 $$\begin{aligned} U(n)\times {\text {Lag}}(n)\longrightarrow {\text {Lag}}(n). \end{aligned}$$

An essential property is the transitivity of these actions.

#### Proposition 2

The subgroup *U*(*n*) of ${\text {Sp}}(n)$ (and hence ${\text {Sp}}(n)$ itself) acts transitively on the Lagrangian Grassmannian ${\text {Lag}}(n)$: for any pair $(\ell ,\ell ^{\prime })$ of Lagrangian planes in $({\mathbb {R}}^{2n},\omega )$ there exists $U\in U(n)$ such that $\ell ^{\prime }=U\ell$. In particular every $\ell \in {\text {Lag}} (n)$ can be obtained from $\ell _{X}$ (or from $\ell _{P}$) using a symplectic rotation.

#### Proof

This is proven as follows [11]: let ${\mathcal {B}}=\{e_{1},\ldots ,e_{n}\}$ and ${\mathcal {B}}^{\prime }=\{e_{1}^{\prime },\ldots ,e_{n}^{\prime }\}$ be orthonormal bases of $\ell$ and $\ell ^{\prime }$, respectively. Then ${\mathcal {B}}\cup J{\mathcal {B}}$ and ${\mathcal {B}}^{\prime }\cup J{\mathcal {B}} ^{\prime }$ are bases of ${\mathbb {R}}^{2n}$ which are both orthogonal and symplectic. Let *U* be a linear mapping taking ${\mathcal {B}}\cup J{\mathcal {B}}$ to ${\mathcal {B}}^{\prime }\cup J{\mathcal {B}}^{\prime }$; we then have $\ell ^{\prime }=U\ell$ and $U\in {\text {Sp}}(n)\cap O(2n,{\mathbb {R}})$. $\square$

The action (10) allows to endow ${\text {Lag}}(n)$ with a topology, using the theory of homogeneous spaces. In fact, the subgroup *O*(*n*) of *U*(*n*) consisting of all symplectic matrices

$$\begin{aligned} R= \begin{pmatrix} A &{}\quad 0\\ 0 &{}\quad A \end{pmatrix} A\in O(n,{\mathbb {R}}) \end{aligned}$$

stabilizes $\ell _{P}$ (that is, $R\ell _{P}=\ell _{P}$) hence there is a natural bijection

$$\begin{aligned} U(n)/O(n)\equiv U(n,{\mathbb {C}})/O(n,{\mathbb {R}})\longrightarrow {\text {Lag}}(n) \end{aligned}$$

which allows to identify topologically the coset space *U*(*n*)/*O*(*n*) with the Lagrangian Grassmannian (see [11] for technical details).

Let $(\ell ,\ell ^{\prime })$ be a pair of Lagrangian planes in $({\mathbb {R}} ^{2n},\omega )$ such that $\ell \cap \ell ^{\prime }=0$. Since the dimensions of $\ell$ and $\ell ^{\prime }$ are *n* this is equivalent to $\ell \oplus \ell ^{\prime }={\mathbb {R}}^{2n}$. We will call $(\ell ,\ell ^{\prime })$ a *Lagrangian frame*. We will use the notation

11 $$\begin{aligned} \ell _{X}={\mathbb {R}}_{x}^{n}\times 0\text { and }\ell _{P}=0\times {\mathbb {R}}_{p}^{n} \end{aligned}$$

and call the spaces $\ell _{X}$ and $\ell _{P}$ the *position* and *momentum planes*; Clearly $(\ell _{X},\ell _{P})$ is a Lagrangian frame (we will call it the“canonical frame”). We will denote the space of all Lagrangian frames ${\text {Lag}} _{0}^{2}(n)$. Thus:

12 $$\begin{aligned} {\text {Lag}}_{0}^{2}(n)=\{(\ell ,\ell ^{\prime })\in {\text {Lag}}^{2}(n):\ell \cap \ell ^{\prime }=\{0\}\} \end{aligned}$$

where ${\text {Lag}}^{2}(n)$ denotes the Cartesian product ${\text {Lag}}(n)\times {\text {Lag}}(n)$.

A crucial property is that the symplectic group ${\text {Sp}}(n)$ acts transitively on the set of all Lagrangian frames [11]. Because of the importance of this result we prove it here:

#### Proposition 3

The group ${\text {Sp}}(n)$ acts transitively on the set of all Lagrangian frames: if $(\ell _{1},\ell _{1}^{\prime })$ and $(\ell _{2},\ell _{2}^{\prime })$ are in ${\text {Lag}}_{0}^{2}(n)$ then there exits $S\in {\text {Sp}}(n)$ such that $(\ell _{2},\ell _{2}^{\prime })=(S\ell _{1},S\ell _{1}^{\prime })$.

#### Proof

Choose a basis $\mathcal {B=}\{e_{11},\ldots ,e_{1n}\}$ of $\ell _{1}$ and a basis ${\mathcal {B}}^{\prime }=\{f_{11},\ldots ,f_{1n}\}$ of $\ell _{1}^{\prime }$ such that $\{e_{1i},f_{1j}\}_{1\le i,j\le n}$ is a symplectic basis of $({\mathbb {R}} _{z}^{2n},\omega )$ (i.e. $\omega (e_{i1},e_{j1})=\omega (f_{i1},f_{j1})=0$ and $\omega (f_{i1},e_{j1})=\delta _{ij}$ for all $i,j=1,\ldots ,n$). Similarly choose bases of $\ell _{2}$ and $\ell _{2}^{\prime }$ whose union $\{e_{2i},f_{2j}\}_{1\le i,j\le n}$ is also a symplectic basis. Define a linear mapping $S:{\mathbb {R}}^{2n}\longrightarrow {\mathbb {R}}^{2n}$ by $S(e_{1i})=e_{2i}$ and $S(f_{1i})=f_{2i}$ for $1\le i\le n$. We have $S\in$ ${\text {Sp}}(n)$ and $(\ell _{2},\ell _{2}^{\prime })=(S\ell _{1},S\ell _{1}^{\prime })$. $\square$

Notice that we cannot replace ${\text {Sp}}(n)$ with *U*(*n*) in the result above. For instance, in the case $n=1$ no rotation will take an arbitrary pair of transverse of lines to another arbitrary pair of transverse lines if they do not form equal angles ($U(1)=SO(2,{\mathbb {R}})$ preserves angles, while ${\text {Sp}}(1)$ does not).

#### Remark 4

It follows from Proposition 3 that every Lagrangian frame in $({\mathbb {R}}^{2n},\omega )$ can be obtained from the canonical frame $(\ell _{X},\ell _{P})$ using a symplectic transformation.

The following property is useful when considering phase space shifts of the origin:

#### Lemma 5

Every phase space point $z_{0}\in {\mathbb {R}}^{2n}$ belongs to at least one Lagrangian plane.

#### Proof

The case $z_{0}=0$ being trivial we assume $z_{0}\ne 0$. Let $e_{1}$ be a normalized vector such that $z_{0}=\lambda e_{1}$ and choose vectors $e_{2},\ldots ,e_{n}$ and $f_{2},\ldots ,f_{n}$ such that $\{e_{1},\ldots ,e_{n} \}\cup \{f_{1},\ldots ,f_{n}\}$ is a symplectic basis of ${\mathbb {R}}^{2n}$ (this is a symplectic variant of the Gram–Schmidt orthonormalization process, see [11] for an explicit construction). The subspace spanned by the set of vectors $\{e_{1},\ldots ,e_{n}\}$ is Lagrangian and contains $z_{0}$. $\square$

### Lagrangian Ellipsoids

Let us identify the position space ellipsoid

$$\begin{aligned} X=\{x\in {\mathbb {R}}_{x}^{n}:Ax\cdot x\le \hbar \} \end{aligned}$$

with the phase space subset

$$\begin{aligned} X=\{z=(x,0):(A\oplus 0)z\cdot z\le \hbar \} \end{aligned}$$

where, by definition,

$$\begin{aligned} A\oplus 0= \begin{pmatrix} A &{}\quad 0_{n\times n}\\ 0_{n\times n} &{}\quad 0_{n\times n} \end{pmatrix}. \end{aligned}$$

The image of *X* by $S\in {\text {Sp}}(n)$ (or by any phase space automorphism) is then

13 $$\begin{aligned} S(X)=\{z:((S^{T})^{-1}(A\oplus 0)S^{-1})z\cdot z\le \hbar \}. \end{aligned}$$

Let us call “quantum blob” [13] the image of the phase space ball $B^{2n}(z_{0},\sqrt{\hbar })$ by a symplectic transformation. The following property shows that every ellipsoid carried by a Lagrangian plane $\ell$ is the intersection $\ell \cap Q$ of that subspace with a quantum blob (or any other phase space ball, for that):

#### Proposition 6

Let $X_{\ell }$ be an *n*-dimensional ellipsoid centered at $z_{0}\in \ell$ and carried by the Lagrangian plane $\ell \in {\text {Lag}}(n)$. There exists $S\in {\text {Sp}}(n)$ such that $X_{\ell }=S(B^{2n}(S^{-1}z_{0},\sqrt{\hbar }))\cap \ell$.

#### Proof

It is sufficient to assume $z_{0}=0$. We first consider the case $\ell =\ell _{X}$, then $X_{\ell _{X}}=\{x:Ax\cdot x\le \hbar \}$ where *A* is a symmetric positive definite matrix. Clearly, $X_{\ell _{X}}$ is the intersection of the phase space ellipsoid

$$\begin{aligned} \Omega =\{(x,p):Ax\cdot x+A^{-1}p\cdot p\le \hslash \} \end{aligned}$$

with $\ell _{X}$, and $\Omega$ is indeed a quantum blob since $\Omega =S(B^{2n}(\sqrt{\hbar }))$ with

14 $$\begin{aligned} S= \begin{pmatrix} A &{}\quad 0\\ 0 &{}\quad A^{-1} \end{pmatrix} \in {\text {Sp}}(n). \end{aligned}$$

Suppose now $\ell$ is an arbitrary Lagrangian plane. In view of Proposition 2 there exists a symplectic rotation $R\in U(n)$ such that $\ell =R\ell _{X}$. The set $X_{\ell _{X}}=R^{-1}(X_{\ell })$ is an ellipsoid in $\ell _{X}$ centered at $z_{0}=0$ and hence $X_{\ell _{X}}=Q\cap \ell _{X}$ for some quantum blob *Q*, and $X_{\ell }=R(X_{\ell _{X}})=(RQ)\cap \ell$ which concludes the proof since *R*(*Q*) is also a quantum blob. $\square$

#### Remark 7

The quantum blob described in the result above is not unique. For instance there exist infinitely many quantum blobs $Q=S(B^{2n}(\sqrt{\hbar }))$ such that $X_{\ell _{X}}=Q\cap \ell _{X}$.

## Lagrangian Polar Duality and Quantum States

### Polar Duality: Review

We begin by briefly recalling the usual notion of polar duality from convex geometry (we are following our presentation in [15]); for the notions of convex geometry we use see for instance [5, 23, 26]). Let *X* be a convex body in configuration space ${\mathbb {R}}_{x}^{n}$ (a convex body is a compact convex set with non-empty interior). We assume in addition that *X* contains 0 in its interior. This is the case if, for instance, *X* is symmetric: $X=-X$. The *polar dual* of *X* is the subset

15 $$\begin{aligned} X^{\hbar }=\{p\in {\mathbb {R}}_{x}^{n}:\sup _{x\in X}(p\cdot x)\le \hbar \} \end{aligned}$$

of the dual space ${\mathbb {R}}_{p}^{n}\equiv ({\mathbb {R}}_{x}^{n})^{*}$. Notice that it trivially follows from the definition that $X^{\hbar }$ is convex and contains 0 in its interior. In the mathematical literature one usually chooses $\hbar =1$, in which case one writes $X^{o}$ for the polar dual; we have $X^{\hbar }=\hbar X^{o}$. The following properties are straightforward:

**Table:** 

*Reflexivity (bipolarity)*:	$(X^{\hbar })^{\hbar }=X$	P1
*Antimonotonicity:*	$X\subset Y\Longrightarrow Y^{\hbar }\subset X^{\hbar }$	P2
*Scaling property*	$A\in GL(n,{\mathbb {R}})\Longrightarrow (AX)^{\hbar }=(A^{T})^{-1}X^{\hbar }$.	P3

In [15] we proved the following elementary properties of polar duality:

*(i)* Let $B_{X}^{n}(R)$ (*resp*. $B_{P}^{n}(R)$) be the ball $\{x:|x|\le R\}$ in ${\mathbb {R}}_{x}^{n}$ (*resp*. $\{p:|p|\le R\}$ in ${\mathbb {R}}_{p}^{n}$). Then

16 $$\begin{aligned} B_{X}^{n}(R)^{\hbar }=B_{P}^{n}(\hbar /R)~. \end{aligned}$$

In particular

17 $$\begin{aligned} B_{X}^{n}(\sqrt{\hbar })^{\hbar }=B_{P}^{n}(\sqrt{\hbar }). \end{aligned}$$

*(ii)* Let *A* be a real invertible and symmetric $n\times n$ matrix and $R>0$. The polar dual of the ellipsoid defined by $Ax\cdot x\le R^{2}$ is given by

18 $$\begin{aligned} \{x:Ax\cdot x\le R^{2}\}^{\hbar }=\{p:A^{-1}p\cdot p\le (\hbar /R)^{2}\} \end{aligned}$$

and hence

19 $$\begin{aligned} \{x:Ax\cdot x\le \hbar \}^{\hbar }=\{p:A^{-1}p\cdot p\le \hbar \}~. \end{aligned}$$

We can easily picture that the polar set $X^{\hbar }$ is “large” when *X* is “small” since *X* and $X^{\hbar }$ are “inversely” related [26]; these sets can also be viewed as Fourier transforms of each other. These qualitative statements, reminiscent of the uncertainty principle, are clarified by the following remarkable property of polar duality, called the *Blaschke–Santaló inequality*: assume that *X* is a symmetric body; then there exists [10] $c>0$ such that

20 $$\begin{aligned} c\le {\text {Vol}}_{n}(X){\text {Vol}}_{n} (X^{\hbar })\le ({\text {Vol}}_{n}(B^{n}(\sqrt{\hbar }))^{2} \end{aligned}$$

where ${\text {Vol}}_{n}$ is the standard Lebesgue measure on ${\mathbb {R}}^{n}$, and equality is attained if and only if $X\subset {\mathbb {R}}_{x}^{n}$ is an ellipsoid centered at the origin The Mahler conjecture (which is still unproven) is that the best constant *c* is $(4\hbar )^{n}/n!$ (see [15]) for a discussion of partial results and references).

### Lagrangian Polar Duality

Let now $(\ell ,\ell ^{\prime })$ be a Lagrangian frame in the symplectic phase space $({\mathbb {R}}^{2n},\omega )$ and $X_{\ell }$ a centrally symmetric convex body in $\ell$ (i.e. $X_{\ell }=-X_{\ell }$). The Lagrangian polar dual $X_{\ell ^{\prime }}^{\hbar }$ of $X_{\ell }$ in $\ell ^{\prime }$ is the subset of $\ell ^{\prime }$ consisting of all $z^{\prime }\in \ell ^{\prime }$ such that

21 $$\begin{aligned} \omega (z^{\prime },z)\le \hbar \text { for all }z\in X_{\ell }; \end{aligned}$$

equivalently, since $X_{\ell }$ is centrally symmetric and $\omega$ antisymmetric,

22 $$\begin{aligned} \omega (z,z^{\prime })\le \hbar \text { for all }z\in X_{\ell }. \end{aligned}$$

The Lagrangian polar dual $X_{\ell ^{\prime }}^{\hbar }$ is also a centrally symmetric body. Suppose in particular that the Lagrangian frame $(\ell ,\ell ^{\prime })$ is the canonical frame $(\ell _{X},\ell _{P})$. Then $z=(x,0)$ and $z^{\prime }=(0,p^{\prime })$ so that condition (21) becomes $p^{\prime }\cdot x\le \hbar$; the notion of Lagrangian polar duality for $(\ell _{X},\ell _{P})$ thus reduces the usual notion of polar duality as described above. It is always possible to reduce Lagrangian polar duality to ordinary polar duality. Recall that the symplectic group acts transitively on the manifold of Lagrangian frames.

#### Proposition 8

Let $(X_{\ell },X_{\ell ^{\prime }}^{\hbar })$ be a dual pair and choose $S\in {\text {Sp}}(n)$ such that $(\ell ,\ell ^{\prime })=S(\ell _{X},\ell _{P})$. Let $X=S^{-1}(X_{\ell })\subset \ell _{X}$. We have $S^{-1}X_{\ell ^{\prime }}^{\hbar }=X^{\hbar }\subset \ell _{P}$. Thus

23 $$\begin{aligned} (X_{\ell },X_{\ell ^{\prime }}^{\hbar })=S(X,X^{\hbar })\,\,\,\, \textit{if}\,\,\,\,(\ell ,\ell ^{\prime })=S(\ell _{X},\ell _{P}) \end{aligned}$$

($X^{\hbar }\subset \ell _{P}$ is the ordinary polar dual of $X\subset \ell _{X}$).

#### Proof

Let $z\in X_{\ell }$ and $z^{\prime }\in X_{\ell ^{\prime }}^{\hbar }$ and define $(x,0)=S^{-1}z$, $(0,p^{\prime })=S^{-1}z^{\prime }$. We have

$$\begin{aligned} p^{\prime }\cdot x=\omega ((x,0);\quad (0,p^{\prime }))=\omega ((S^{-1}z;S^{-1} z^{\prime })=\omega ((z;z^{\prime }) \end{aligned}$$

hence the conditions $\omega (z,z^{\prime })\le \hbar$ and $p^{\prime }\cdot x\le \hbar$ are equivalent. $\square$

The following table summarizes the main properties of Lagrangian polar duality:

**Table:** 

*Reflexivity*:	$(X_{\ell ^{\prime }}^{\hbar })_{\ell }^{\hbar }=X_{\ell }$	LP1
*Antimonotonicity:*	$X_{\ell }\subset Y_{\ell }\Longrightarrow Y_{\ell ^{\prime }}^{\hbar }\subset X_{\ell ^{\prime }}^{\hbar }$	LP2
*Symplectic covariance*:	$S\in {\text {Sp}}(n)\Longrightarrow S(X_{\ell ^{\prime }}^{\hbar })=(SX_{\ell })_{S\ell ^{\prime }}^{\hbar }.$	LP3

The following characteristic property of quantum blobs is also useful:

#### Proposition 9

Let $Q=S(B^{2n}(\sqrt{\hbar }))$ be a centered quantum blob and $(\ell _{X},\ell _{P})\in {\text {Lag}}_{0}^{2}(n)$ the canonical Lagrangian frame. The intersection $Q\cap \ell _{X}$ and the orthogonal projection $\Pi _{\ell _{P} }Q$ are polar dual of each other. We have a similar statement interchanging $\ell _{X}$ and $\ell _{P}$.

#### Proof

We have to show that $Q\cap \ell _{X}$ and $\Pi _{\ell _{P}}Q$ are *n*-dimensional ellipsoids $\{x:Ax\cdot x\le \hbar \}$ and $\{p:Bp\cdot p\le \hbar \}$ such that $AB=I_{n\times n}$. The quantum blob *Q* is represented by the inequality $Gz\cdot z\le \hbar$ where $G=(SS^{T})^{-1}\in {\text {Sp}}(n)$. Writing *G* in block matrix form $$\begin{pmatrix} G_{XX} &{}\quad G_{XP}\\ G_{PX} &{}\quad G_{PP} \end{pmatrix}$$ the following relations hold in view of the symplectic conditions (3), taking into account the symmetry of *G*:

24 $$\begin{aligned} G_{XX}G_{PX}, G_{PX}G_{PP} \textit{ symmetric and } G_{XX} G_{PP}-G_{XP}^{2}=I_{n\times n}. \end{aligned}$$

With this notation we clearly have

$$\begin{aligned} Q\cap \ell _{X}=\{x:G_{XX}x\cdot x\le \hbar \} \end{aligned}$$

while the orthogonal projection $\Pi _{\ell _{P}}Q$ is given by (see [15])

$$\begin{aligned} \Pi _{\ell _{P}}Q=\{p:(G/G_{XX})p\cdot p\le \hbar \} \end{aligned}$$

where $G/G_{XX}$ is the Schur complement

$$\begin{aligned} G/G_{XX}=G_{PP}-G_{PX}G_{XX}^{-1}G_{XP}. \end{aligned}$$

To prove the proposition it therefore suffices to show that

$$\begin{aligned} G_{XX}(G_{PP}-G_{PX}G_{XX}^{-1}G_{XP})=I_{n\times n} \end{aligned}$$

but this follows from the relations (24) which in particular imply that $G_{PX}G_{XX}^{-1}=G_{XX}^{-1}G_{PX}$:

$$\begin{aligned} G_{XX}(G_{PP}-G_{PX}G_{XX}^{-1}G_{XP})&=G_{XX}G_{PP}-G_{XX}(G_{PX} G_{XX}^{-1})G_{XP})\\&=G_{XX}G_{PP}-G_{XP}^{2})=I_{n\times n}. \end{aligned}$$

$$\square$$

## Lagrangian Quantum States

### Definition of a Lagrangian Quantum State

The definition of quantum states we are giving here generalizes the Definition 3 in [15].

#### Definition 10

(*Centered case*)Let $(\ell ,\ell ^{\prime })\in {\text {Lag}} _{0}^{2}(n)$ be a Lagrangian frame and $X_{\ell }$ be an ellipsoid with center 0 carried by $\ell$. We call the product $X_{\ell }\times X_{\ell ^{\prime } }^{\hbar }$ the Lagrangian quantum state in ${\mathbb {R}}^{2n}$ associated with the frame $(\ell ,\ell ^{\prime })$ and the ellipsoid $X_{\ell }$ and we set

$$\begin{aligned} {\text {Quant}}_{0}(n)=\{X_{\ell }\times X_{\ell ^{\prime } }^{\hbar }:(\ell ,\ell ^{\prime })\in {\text {Lag}}_{0}^{2}(n)\}. \end{aligned}$$

The elements of ${\text {Quant}}_{0}(1)$ are parallelograms with area $4\hbar$ in the phase plane, while ${\text {Quant}}_{0}(2)$ consist of products of two dual plane ellipses. The simplest example of a state in 2*n*-dimensional phase space is what we call the “fiducial state”, defined by

25 $$\begin{aligned} X_{\ell _{X}}\times X_{\ell _{P}}^{\hbar }=B_{X}^{n}(\sqrt{\hbar })\times B_{P}^{n}(\sqrt{\hbar }). \end{aligned}$$

To define a quantum state when the ellipsoid $X_{\ell }$ has center $z_{0} \ne 0$ some care is needed. Consider for example, for $\hbar =1$, the polar dual $X^{1}$ of the disk $X=B^{2}((a,0),1)$ in the *x*, *y* plane, where $0\le a<1$. It is the ellipse defined by [5]

26 $$\begin{aligned} (1-a^{2})^{2}\left( p_{x}+\frac{a}{1-a^{2}}\right) ^{2}+(1-a^{2})p_{y} ^{2}\le 1 \end{aligned}$$

and its area $\pi /(1-a^{2})$ becomes arbitrarily large when *a* gets close to one. To avoid this unwanted lack of stability we proceed as follows: suppose the ellipsoid $X_{\ell }(z_{0})$ is centered at some $z_{0}\in \ell$ and consider the translate $X_{\ell }=T(-z_{0})X_{\ell }(z_{0})$ (it is the set of all $z-z_{0}$ for $z\in X_{\ell }(z_{0})$). Since $X_{\ell }$ has center 0 we can define as usual its Lagrangian polar $X_{\ell ^{\prime }}^{\hbar }$, and by definition this will be the Lagrangian polar dual of $X_{\ell }(z_{0})$. This procedure, has been generalized by Santaló [24] to arbitrary convex bodies, but is much more complicated in this case. This leads to the following extension of Definition 10:

#### Definition 11

(*General case*)Let $(\ell ,\ell ^{\prime })\in {\text {Lag}} _{0}^{2}(n)$ and $(z_{0},z_{0}^{\prime })\in \mathbb {\ell \times \ell }^{\prime }$ (cf. Lemma 5). Let $X_{\ell }(z_{0})=T(z_{0})X_{\ell }$ be an ellipsoid carried by $\ell$ and centered at $z_{0}$. The Lagrangian quantum state associated with $(\ell ,\ell ^{\prime },z_{0},z_{0}^{\prime })$ and $X_{\ell }$ is the product

27 $$\begin{aligned} X_{\ell }(z_{0})\times (X_{\ell }(z_{0})-z_{0})_{\ell ^{\prime }}^{\hbar } +z_{0}^{\prime })=X_{\ell }(z_{0})\times X_{\ell ^{\prime }}^{\hbar }(z_{0} ^{\prime }) \end{aligned}$$

where we write $X_{\ell ^{\prime }}^{\hbar }(z_{0}^{\prime })=T(z_{0}^{\prime })X_{\ell ^{\prime }}^{\hbar }$. We denote ${\text {Quant}}(n)$ the set of all such quantum states.

Here is a basic example:

#### Example 12

Let $$z_{0}=(x_{0},0)$$, $$z_{0}^{\prime }=(0,p_{0})$$, $$\ell =\ell _{X}$$, $$\ell ^{\prime }=\ell _{P}$$, and

$$\begin{aligned} X_{\ell }(z_{0})=T(x_{0},0)(B_{X}^{n}(\sqrt{\hbar })\times 0)=B_{X}^{n} (x_{0},\sqrt{\hbar })\times 0. \end{aligned}$$

We have $(B_{X}^{n}(\sqrt{\hbar })\times 0)_{\ell _{P}}^{\hbar }=0\times B_{P} ^{n}(\sqrt{\hbar })$ hence the state is

$$\begin{aligned} (B_{X}^{n}(x_{0},\sqrt{\hbar })\times 0)\times (0\times B_{P}^{n}(p_{0},\sqrt{\hbar }))\equiv B_{X}^{n}(x_{0},\sqrt{\hbar })\times B_{P}^{n} (p_{0},\sqrt{\hbar }). \end{aligned}$$

In classical mechanics the phase space ${\mathbb {R}}_{x}^{n}\times {\mathbb {R}} _{p}^{n}$ can be viewed as a fiber bundle over the configuration space ${\mathbb {R}}_{x}^{n}$ using the projection $\pi _{x}(x,p)=x$; the fiber is then just the momentum space ${\mathbb {R}}_{p}^{n}$. In the case of Lagrangian quantum states we have a similar situation replacing the points in configuration space with ellipsoids (“pointillisme”). Let ${\mathcal{E}\ell \ell (}{\mathbb {R}}_{x}^{n})$ be the set of all ellipsoids in $\ell _{X}={\mathbb {R}}_{x}^{n}$; a typical element of ${\mathcal{E}\ell \ell (}{\mathbb {R}}_{x}^{n})$ is the set of all *x* such that $A(x-x_{0})\cdot (x-x_{0})\le \hbar$. For instance, $B_{X}^{n} (x_{0},\sqrt{\hbar })\in \mathcalligra {E\ell \ell (}{\mathbb {R}}_{x}^{n})$. Let us now work using the canonical Lagrangian frame $(\ell _{X},\ell _{P})$ and denote by ${\text {Quant}}_{\text{can}}(n)\subset {\text {Quant}} (n)$ the set of quantum states $X(x_{0},0)\times X^{\hbar }(0,p_{0})$ where $X(x_{0},0)\subset \ell _{X}$ and $X^{\hbar }(0,p_{0})\subset \ell _{P}$ is in $\mathcalligra {E\ell \ell (}{\mathbb {R}}_{p}^{n})$. We define a projection $\pi _{\text{can}}:{\text {Quant}}_{\text{can} }(n)\longrightarrow \mathcalligra {E\ell \ell (}{\mathbb {R}}_{x}^{n})$ by

$$\begin{aligned} \pi _{\text{can}}(X(x_{0},0)\times X^{\hbar }(0,p_{0}))=X(x_{0},0) \end{aligned}$$

which defines a vector bundle structure on ${\text {Quant}}_{\text{can}}(n)$. The fiber over $X(x_{0},0)\in \mathcalligra {E\ell \ell (}{\mathbb {R}}_{x}^{n})$ is

$$\begin{aligned} \pi _{\text{can}}^{-1}(X(x_{0},0))=\{X(x_{0},0)\times X^{\hbar } (0,p_{0}):p_{0}\in {\mathbb {R}}_{p}^{n}\} \end{aligned}$$

so we have the identification

$$\begin{aligned} \pi _{\text{can}}^{-1}(X(x_{0},0))\equiv X(x_{0},0)\times \mathcalligra {E\ell \ell (}{\mathbb {R}}_{p}^{n}). \end{aligned}$$

### Symplectic Actions on ${\text {Quant}}_{0}(n)$

As expected, elliptic quantum states behave well under linear or affine symplectic transformations. Recall from Proposition 8 that for every dual pair $(X_{\ell },X_{\ell ^{\prime }}^{\hbar })$ there exists $S\in {\text {Sp}}(n)$ such that $(\ell ,\ell ^{\prime })=S(\ell _{X},\ell _{P})$ and $(X_{\ell },X_{\ell ^{\prime }}^{\hbar })=S(X,X^{\hbar })$. Every quantum state $X_{\ell }\times X_{\ell ^{\prime }}^{\hbar }$ is thus the image by some $S\in {\text {Sp}}(n)$ of a quantum state $X\times X^{\hbar }\subset \ell _{X}\times \ell _{P}$ associated with the canonical Lagrangian frame. The action of ${\text {Sp}}(n)$ on ${\text {Quant}}_{0}(n)$ is thus naturally defined by the formula

28 $$\begin{aligned} S^{\prime }(X_{\ell }\times X_{\ell ^{\prime }}^{\hbar })=S^{\prime }S(X\times X^{\hbar })\subset S^{\prime }S\ell _{X}\times S^{\prime }S\ell _{P}. \end{aligned}$$

We have a similar definition for the action of ${\text {Sp}}(n)$ on ${\text {Quant}}(n)$. We define the action of $S^{\prime }\in {\text {Sp}}(n)$ on the state $X_{\ell }(z_{0})\times X_{\ell ^{\prime } }^{\hbar }(z_{0}^{\prime })$ by

29 $$\begin{aligned} S^{\prime }(X_{\ell }(z_{0})\times X_{\ell ^{\prime }}^{\hbar }(z_{0}^{\prime }))=T(S^{\prime }z_{0})SX_{\ell }\times T(S^{\prime }z_{0}^{\prime } )(SX)_{\ell ^{\prime }}^{\hbar }. \end{aligned}$$

This can be rewritten, taking (28) into account,

30 $$\begin{aligned} S^{\prime }(X_{\ell }(z_{0})\times X_{\ell ^{\prime }}^{\hbar }(z_{0}^{\prime }))=(S^{\prime }SX)(S^{\prime }z_{0})\times (S^{\prime }SX^{\hbar })(S^{\prime }z_{0}^{\prime }). \end{aligned}$$

#### Proposition 13

(i) The symplectic action

31 $$\begin{aligned} {\text {Sp}}(n)\times {\text {Quant}}_{0} (n)\longrightarrow {\text {Quant}}_{0}(n) \end{aligned}$$

defined by (28) is transitive. In particular, for every state $X_{\ell }\times X_{\ell ^{\prime }}^{\hbar }$ there exists $S\in {\text {Sp}}(n)$ such that

32 $$\begin{aligned} X_{\ell }\times X_{\ell ^{\prime }}^{\hbar }=S(B_{X}^{n}(\sqrt{\hbar })\times B_{P}^{n}(\sqrt{\hbar })) \end{aligned}$$

(that *S* is not unique: see Remark 7). (ii) The symplectic action

33 $$\begin{aligned} {\text {Sp}}(n)\times {\text {Quant}}(n)\longrightarrow {\text {Quant}}(n) \end{aligned}$$

defined by (29) is also transitive, and there exists $S\in {\text {Sp}}(n)$ such that

34 $$\begin{aligned} X_{\ell }(z_{0})\times X_{\ell ^{\prime }}^{\hbar }(z_{0}^{\prime })=S(B_{X} ^{n}(x_{0},\sqrt{\hbar })\times B_{P}^{n}(p_{0},\sqrt{\hbar })) \end{aligned}$$

where $x_{0}$ and $p_{0}$ are defined by: $(x_{0},0)=S^{-1}z_{0}$ and $(0,p_{0})=S^{-1}z_{0}^{\prime }$.

#### Proof

To prove part *(i)* it is sufficient to show that there exists $S\in {\text {Sp}}(n)$ such that (32) holds. Let now $S\in {\text {Sp}}(n)$ be such that $(\ell ,\ell ^{\prime })=S(\ell _{X},\ell _{P})$ and $(X_{\ell },X_{\ell ^{\prime }}^{\hbar })=S(X,X^{\hbar })$. There exists a symmetric positive definite matrix *A* such that ellipsoid *X* is $A^{-1/2}(B_{X}^{n}(\sqrt{\hbar }))$ hence $X^{\hbar }=A^{1/2}(B_{X}^{n} (\sqrt{\hbar }))$ and

$$\begin{aligned} X\times X^{\hbar }=M_{A^{1/2}}(B_{X}^{n}(\sqrt{\hbar })\times B_{P}^{n} (\sqrt{\hbar })) \end{aligned}$$

where $$M_{A^{1/2}}= \begin{pmatrix} A^{1/2} &{}\quad 0\\ 0 &{}\quad A^{-1/2} \end{pmatrix} \in {\text {Sp}}(n)$$ so that we have

$$\begin{aligned} (X_{\ell },X_{\ell ^{\prime }}^{\hbar })=SM_{A^{1/2}}(B_{X}^{n}(\sqrt{\hbar })\times B_{P}^{n}(\sqrt{\hbar })) \end{aligned}$$

which was to be proven. Part *(ii)* is proven in a similar way. $\square$

### ${\text {Quant}}_{0}(n)$ as a Homogeneous Space

Proposition 13 leads a topological identification of ${\text {Quant}}_{0}(n)$ with the homogeneous space ${\text {Sp}} (n)/O(n)$. We begin by noting that the “fiducial quantum state” $B_{X}^{n}(\sqrt{\hbar })\times B_{P}^{n}(\sqrt{\hbar })$ is invariant by the action of the subgroup *O*(*n*) of *U*(*n*) consisting of all matrices $$M_{H}= \begin{pmatrix} H &{}\quad 0\\ 0 &{}\quad H \end{pmatrix}$$ with $H\in O(n,{\mathbb {R}})$.

#### Remark 14

The quotient ${\text {Sp}}(n)/U(n)$ (which is “smaller” than ${\text {Sp}}(n)/O(n)$) can be identified with the set of Wigner transforms of Gaussian wavepackets [21, formula (8.12)]. This shows that ${\text {Quant}}_{0}(n)$ contains more information than the Gaussian wavepackets which we will study below.

Let us state things in a more precise way. We first note that the “orthogonal symplectic group” *O*(*n*) is the largest subgroup of ${\text {Sp}}(n)$ such that

$$\begin{aligned} S(B_{X}^{n}(\sqrt{\hbar })\times B_{P}^{n}(\sqrt{\hbar }))=B_{X}^{n}(\sqrt{\hbar })\times B_{P}^{n}(\sqrt{\hbar }), \end{aligned}$$

i.e. *O*(*n*) is the stabilizer (or isotropy subgroup) of the action of ${\text {Sp}}(n)$ on $B_{X}^{n}(\sqrt{\hbar })\times B_{P}^{n} (\sqrt{\hbar })$ (we are identifying, as usual, $B_{X}^{n}(\sqrt{\hbar } )\times 0\subset \ell _{X}$ with $B_{X}^{n}(\sqrt{\hbar })$ and $0\times B_{X} ^{n}(\sqrt{\hbar })\times \subset \ell _{P}$ with $B_{P}^{n}(\sqrt{\hbar })$. To see this it suffices to note that if $S(B_{X}^{n}(\sqrt{\hbar }))=B_{X} ^{n}(\sqrt{\hbar })$ and similarly $S(B_{P}^{n}(\sqrt{\hbar }))=B_{P}^{n} (\sqrt{\hbar })$ then we must have, by homogeneity, $S\ell _{X}=\ell _{X}$ and $S\ell _{P}=\ell _{P}$, hence we must have $$S= \begin{pmatrix} H &{}\quad 0\\ 0 &{}\quad H \end{pmatrix}$$ for some $H\in O(n)$. Since ${\text {Sp}}(n)$ is a classical Lie group and *O*(*n*) is a closed subgroup it follows from the theory of homogeneous spaces that we have the identification

35 $$\begin{aligned} {\text {Quant}}_{0}(n)\equiv {\text {Sp}}(n)/O(n) \end{aligned}$$

which allows to define a topology on ${\text {Quant}}_{0}(n)$ and hence a fiber bundle [25]

$$\begin{aligned} {\mathcal {F}}=({\text {Sp}}(n),{\text {Quant}}_{0} (n),\pi _{0}^{{\text {Quant}}},O(n)) \end{aligned}$$

with projection

$$\begin{aligned} \pi _{0}^{{\text {Quant}}}:{\text {Sp}}(n)\longrightarrow {\text {Quant}}_{0}(n). \end{aligned}$$

#### Remark 15

The complex structure rotation $J:(x,p)\longmapsto (p,-x)$ also fixes the Lagrangian product of two same size balls, but does not belong to the group *O*(*n*). On the analytical level *J* plays the role of a Fourier transform.

## ${\text {Quant}}(n)$ and Gaussian Wavepackets

In this section we identify a subset of ${\text {Quant}}(n)$ with the set of all Gaussian wavepackets.

### John and Löwner Ellipsoids

There is a vast literature on the Löwner and John ellipsoids of a convex body; a classical reference is [6]. Let *X* be a convex body in any Euclidean space ${\mathbb {R}}^{n}$. The Löwner ellipsoid $X_{\mathrm {L\ddot{o}wner}}$ of *X* *is the unique ellipsoid in* ${\mathbb {R}}^{n}$ *with minimum volume containing* *X* and the John ellipsoid $X_{\text{John}}$ *is the unique ellipsoid in* ${\mathbb {R}}^{n}$ *with maximum volume contained in* *X*. If *A* is an invertible linear mapping then

36 $$\begin{aligned} (A(X))_{\text{L}\ddot{\text{o}}\text{wner}}=A(X_{\text{L}\ddot{\text{o}}\text{wner}}), (A(X))_{\text{John}}=A(X_{\text{John}}) \end{aligned}$$

Not so surprisingly, if *X* is a centrally symmetric convex body, then $X_{\text{John}}$ and $X_{\mathrm {L\ddot{o}wner}}$ are polar duals of each other in the following sense [5]:

37 $$\begin{aligned} (X_{\text{John}})^{\hbar }=(X^{\hbar })_{\text{L}\ddot{\text{o}}\text{wner}}, (X_{\text{L}\ddot{\text{o}}\text{wner}})^{\hbar }=(X^{\hbar })_{\text{John}}. \end{aligned}$$

This property extends to Lagrangian polar duality. Let $(\ell ,\ell ^{\prime })$ be a Lagrangian frame and $(X_{\ell },X_{\ell ^{\prime }}^{\hbar })$ a dual pair of centered convex bodies. Then

38 $$\begin{aligned} ((X_{\ell })_{\text{John}})_{\ell ^{\prime }}^{\hbar }=(X_{\ell ^{\prime }} ^{\hbar })_{\text{L}\ddot{\text{o}}\text{wner}}, ((X_{\ell })_{\text{L}\ddot{\text{o}}\text{wner}})_{\ell ^{\prime }}^{\hbar }=(X_{\ell ^{\prime }}^{\hbar })_{\text{John} }. \end{aligned}$$

The following particular case will be very important for what follows. We denote $B_{X}^{n}(R)$ (resp. $B_{P}^{n}(R)$) the ball $|x|\le R$ (resp. $|p|\le R$) in position (resp. momentum) space.

#### Proposition 16

The John ellipsoid of $B_{X}^{n}(R)\times B_{P}^{n}(R)$ is $B^{2n}(R)$. In particular

39 $$\begin{aligned} \left( B_{X}^{n}(\sqrt{\hbar })\times B_{P}^{n}(\sqrt{\hbar })\right) _{\text{John}}=B^{2n}(\sqrt{\hbar }). \end{aligned}$$

#### Proof

The inclusion

40 $$\begin{aligned} B^{2n}(R)\subset B_{X}^{n}(R)\times B_{P}^{n}(R) \end{aligned}$$

is obvious, and we cannot have

$$\begin{aligned} B^{2n}(R^{\prime })\subset B_{X}^{n}(R)\times B_{P}^{n}(R) \end{aligned}$$

if $R^{\prime }>R$. Assume now that the John ellipsoid $\Omega _{\text{John}}$ of $\Omega =B_{X}^{n}(R)\times B_{P}^{n}(R)$ is defined by $Ax^{2} +Bxp+Cp^{2}\le R^{2}$ where $A,C>0$ and *B* are real $n\times n$ matrices. Since $\Omega$ is invariant by the transformation $(x,p)\longmapsto (p,x)$ so is $\Omega _{\text{John}}$ and we must thus have $A=C$ and $B=B^{T}$. Similarly, $\Omega$ being invariant by the partial reflection $(x,p)\longmapsto (-x,p)$ we get $B=0$ so $\Omega _{\text{John}}$ is defined by $Ax^{2}+Ap^{2}\le R^{2}$. We next observe that $\Omega$ and hence $\Omega _{\text{John}}$ are invariant under the symplectic transformations $(x,p)\longmapsto (Hx,HP)$ where $H\in O(n,{\mathbb {R}})$ so we must have $AH=HA$ for all $H\in O(n,{\mathbb {R}})$, but this is only possible if $A=\lambda I_{n\times n}$ for some $\lambda \in {\mathbb {R}}$. The John ellipsoid is thus of the type $B^{2n}(R/\sqrt{\lambda })$ for some $\lambda \ge 1$ and this concludes the proof in view of (40) since the case $\lambda >R^{2}$ is excluded. $\square$

### Gaussian Wavepackets and Their Wigner Transforms

Recall [14] that the Wigner transform of a square integrable function $\psi$ on ${\mathbb {R}}_{x}^{n}$ is defined by the absolutely convergent integral

41 $$\begin{aligned} W\psi (x,p)=\left( \tfrac{1}{2\pi \hbar }\right) ^{n}\int e^{-\frac{i}{\hbar }py}\psi (x+\tfrac{1}{2}y)\psi ^{*}(x-\tfrac{1}{2}y)d^{n}y. \end{aligned}$$

The Wigner transform is a real function which can take negative values (except when $\psi$ is a Gaussian). We recall the “marginal properties” of the Wigner transform: if both $\psi$ and its Fourier transform

$$\begin{aligned} \widehat{\psi }(p)=F\psi (p)=\left( \tfrac{1}{2\pi \hbar }\right) ^{n/2}\int e^{-\frac{1}{\hbar }p\cdot x}\psi (x)d^{n}x \end{aligned}$$

are in $L^{1}({\mathbb {R}}_{x}^{n})\cap L^{2}({\mathbb {R}}_{x}^{n})$ then

42 $$\begin{aligned} \int W\psi (x,p)d^{n}p&=|\psi (x)|^{2} \end{aligned}$$

43 $$\begin{aligned} \int W\psi (x,p)d^{n}x&=|F\psi (p)|^{2}. \end{aligned}$$

These relations imply that

44 $$\begin{aligned} \int W\psi (x,p)d^{n}pd^{n}x=||\psi ||_{L^{2}} \end{aligned}$$

so that if $\psi$ is normalized to one then the integral of $W\psi$ over all of phase space is equal to one. These properties motivate the interpretation of the Wigner transform as a quasi-probability density.

A crucial fact is that the Wigner transform enjoys the property of symplectic covariance [11, 14], that is, we have for every $S\in {\text {Sp}}(n)$,

45 $$\begin{aligned} W\psi (S^{-1}z)=W(\widehat{S}\psi )(z) \end{aligned}$$

where $\widehat{S}$ is anyone of the two metaplectic operators covering *S*. This property is instrumental in proving the symplectic covariance of Weyl quantization, and implies that the metaplectic group acts transitively on the Gaussian wavepackets we define below.

Following our work in [12] we introduced in [13] the notion of “quantum blob”. Their properties were detailed in our *Phys. Reps.* paper [17] with F. Luef. A quantum blob is the image of a phase space ball $B^{2n}(z_{0},\sqrt{\hbar }):|z-z_{0}|\le \sqrt{\hbar }$ by some $S\in {\text {Sp}}(n)$. it can be viewed as the smallest phase space unit compatible with the uncertainty principle expressed in terms of variances and covariances (for a discussion of the relevance of the use of standard deviations to formulate the uncertainty relations see [20]). It turns out that there is a canonical correspondence between quantum blobs and Gaussian wavepackets

46 $$\begin{aligned} \psi _{AB}(x)=e^{i\gamma }\left( \tfrac{1}{\pi \hbar }\right) ^{n/4}(\det A)^{1/4}e^{-\tfrac{1}{2\hbar }(A+iB)x\cdot x} \end{aligned}$$

and their displacements $\psi _{AB,z_{0}}=\widehat{T}(z_{0})\psi _{AB}$ by the Heisenberg–Weyl operator $\widehat{T}(z_{0})$ [11, 21]. In (46) *A* and *B* are real symmetric $n\times n$ matrices with *A* positive definite and $\gamma \in R$ an arbitrary constant phase; we will not care about the value of this phase factor since we will be dealing with the properties of the quantum states $|\psi _{AB}\rangle$. When $A=I$ (the identity), $B=0$, and $\gamma =0$ the function $\psi _{AB}$ reduces to the “fiducial coherent state” (we are using the terminology in [21]):

47 $$\begin{aligned} \phi _{0}(x)=(\pi \hbar )^{-n/4}e^{-|x|^{2}/2\hbar }. \end{aligned}$$

It turns out that every Gaussian wavepacket (46) can be obtained from the fiducial state by using metaplectic operators.

We will denote by ${\text {Gauss}}(n)$ the set of all Gaussian wavepackets $\widehat{T}(z_{0})\psi _{AB}$, and by ${\text {Gauss}} _{0}(n)$ the subset consisting of centered wavepackets. One shows [7, 11], using the symplectic covariance formula (45), that the Wigner transform of $\widehat{T}(z_{0})\psi _{AB}$ is the phase space Gaussian

48 $$\begin{aligned} W\psi _{AB}(z)=(\pi \hbar )^{-n}e^{-\tfrac{1}{\hbar }G(z-z_{0})\cdot (z-z_{0})} \end{aligned}$$

where *G* is the positive definite symmetric and symplectic $2n\times 2n$ matrix

49 $$\begin{aligned} G=(S_{AB}S_{AB}^{T})^{-1}, S_{AB}= \begin{pmatrix} A^{-1/2} &{}\quad 0\\ -BA^{-1/2} &{}\quad A^{1/2} \end{pmatrix}. \end{aligned}$$

Let us denote by ${\text {QB}}(n)$ the set of all quantum blobs $S(B^{2n}(z_{0},\sqrt{\hbar }))$, $S\in {\text {Sp}}(n)$ and by ${\text {QB}}_{0}(n)$ the subset consisting of all centered quantum blobs $S(B^{2n}(\sqrt{\hbar }))$. Recall that ${\text {Gauss}}(n)$ is the set of all Gaussian states $\widehat{T}(z_{0})\psi _{AB}$.

#### Proposition 17

(i) There is a bijective correspondence ${\text {Gauss}} (n)\longleftrightarrow {\text {QB}}(n)$; it is defined by

$$\begin{aligned} \widehat{T}(z_{0})\psi _{AB}\longrightarrow T(z_{0})S_{AB}(B^{2n}(\sqrt{\hbar })). \end{aligned}$$

where $T(z_{0})$ is the phase space translation $z\longmapsto z+z_{0}$ and $S_{AB}\in {\text {Sp}}(n)$ is defined by (48) and (49). (ii) The transitive action of ${\text {Sp}}(n)$ on the set ${\text {QB}}_{0}(n)$ of centered quantum blobs induces a transitive action of ${\text {Mp}}(n)$ on ${\text {Gauss}}_{0}(n).$ More generally the transitive action of the inhomogeneous symplectic group ${\text {ISp}}(n)$ on ${\text {QB}}(n)$ induces a transitive action of ${\text {IMp}}(n)$ on ${\text {Gauss}}(n)$.

#### Proof

(i) In view of the discussion above the Wigner transform associates to $\widehat{T}(z_{0})\psi _{AB}$ the phase space ellipsoid

$$\begin{aligned} Q=\{z:G_{AB}(z-z_{0})\cdot (z-z_{0})\le \hbar \} \end{aligned}$$

where $G=(S_{AB}S_{AB}^{T})^{-1}$ hence *Q* is the quantum blob $T(z_{0} )S_{AB}(B^{2n}(\sqrt{\hbar }))$. Let us show that, conversely, every quantum blob is is obtained from a unique state $|\widehat{T}(z_{0})\psi _{AB}\rangle$. Let $Q=T(z_{0})S(B^{2n}(\sqrt{\hbar }))$be a quantum blob, that is

$$\begin{aligned} Q=\{z:G(z-z_{0})\cdot (z-z_{0})\le \hbar \}, G=(SS^{T} )^{-1}. \end{aligned}$$

To *Q* we associate the function $\psi$ with Wigner transform

$$\begin{aligned} W\psi (z)=(\pi \hbar )^{-n}e^{-\tfrac{1}{\hbar }G(z-z_{0})\cdot (z-z_{0})}. \end{aligned}$$

We have

$$\begin{aligned} W\psi (S(z+S^{-1}z_{0}))=(\pi \hbar )^{-n}e^{-\tfrac{1}{\hbar }|z|^{2}}=W\phi _{0}(z) \end{aligned}$$

hence, by the symplectic covariance formula (45),

$$\begin{aligned} W(\widehat{S}\psi )(z)=W\phi _{0}(z-S^{-1}z_{0})=W(\widehat{T}(S^{-1}z_{0} )\phi _{0})(z) \end{aligned}$$

where $\widehat{S}\in {\text {Mp}}(n)$ covers *S*. It follows that we have

$$\begin{aligned} \widehat{S}\psi (x)=e^{i\gamma }\widehat{T}(S^{-1}z_{0})\phi _{0}(x) \end{aligned}$$

that is

$$\begin{aligned} \psi (x)=e^{i\gamma }\widehat{S}\widehat{T}(S^{-1}z_{0})\phi _{0}(x)=e^{i\gamma }\widehat{T}(z_{0})\widehat{S}\phi _{0}(x) \end{aligned}$$

so that $\psi =e^{i\gamma }\widehat{T}(z_{0})\psi _{A,B}$ for some (uniquely defined) matrices *A* and *B*. (ii). see [11]. $\square$

For a detailed study of the correspondence ${\text {Gauss}} (n)\longleftrightarrow {\text {QB}}(n)$ see [13, 17].

### Construction of a Quantum Gaussian Space

Consider first the very simple case where *X* is the ball $B_{X}^{n} (\sqrt{\hbar })$ whose polar dual is $X^{\hbar }=B_{P}^{n}(\sqrt{\hbar })$. The corresponding elliptic quantum state is the product $B_{X}^{n}(\sqrt{\hbar })\times B_{P}^{n}(\sqrt{\hbar })$. In view of Proposition 16 the John ellipsoid of this state is $B^{2n}(\sqrt{\hbar })$, and to the latter corresponds the fiducial coherent state $\phi _{0}(x)=(\pi \hbar )^{-n/4} e^{-|x|^{2}/2\hbar }$. Slightly more generally, let *U* be a symplectic rotation and define a Lagrangian frame $(\ell ,\ell ^{\prime })$ by $\ell =U\ell _{X}$ and $\ell ^{\prime }=U\ell _{P}$. Identifying $B_{X}^{n}(\sqrt{\hbar })$ with $B_{X}^{n}(\sqrt{\hbar })\times 0\subset \ell _{X}$ the rotation *U* takes this set to $U(B_{X}^{n}(\sqrt{\hbar })\times 0)\subset \ell$ and, similarly, $U(B_{P}^{n}(\sqrt{\hbar })\times 0)\subset \ell ^{\prime }$. The state $B_{X}^{n}(\sqrt{\hbar })\times B_{P}^{n}(\sqrt{\hbar })$ is replaced with $U(B_{X}^{n}(\sqrt{\hbar })\times B_{P}^{n}(\sqrt{\hbar }))$ whose John ellipsoid is, by rotational symmetry,

$$\begin{aligned} \left( U(B_{X}^{n}(\sqrt{\hbar })\times B_{P}^{n}(\sqrt{\hbar }))\right) _{\text{John}}=U(B^{2n}(\sqrt{\hbar }))=B^{2n}(\sqrt{\hbar }) \end{aligned}$$

in view of the linear transformation property (36). The states $B_{X}^{n}(\sqrt{\hbar })\times B_{P}^{n}(\sqrt{\hbar }$ and $U(B_{X}^{n} (\sqrt{\hbar })\times B_{P}^{n}(\sqrt{\hbar }))$ thus have the *same* John ellipsoid, and to both states thus corresponds the fiducial Gaussian wavepacket $\phi _{0}$. From the Wigner transform point of view, this property just reflects the rotational invariance of $\phi _{0}$: we have

$$\begin{aligned} W\phi _{0}(Uz)=(\pi \hbar )^{-n}e^{-\frac{1}{\hbar }Uz\cdot Uz}=(\pi \hbar )^{-n}e^{-\frac{1}{\hbar }z\cdot z}=W\phi _{0}(z). \end{aligned}$$

Consider next the slightly more general case where *X* is the ellipsoid

$$\begin{aligned} X=\{x:Ax\cdot x\le \hbar \}=A^{-1/2}(B_{X}^{n}(\sqrt{\hbar })) \end{aligned}$$

with $$A=A^{T}>0$$; hence

$$\begin{aligned} X^{\hbar }=\{p:A^{-1}p\cdot p\le \hbar \}=A^{1/2}(B_{P}^{n}(\sqrt{\hbar })) \end{aligned}$$

and the corresponding quantum state is then

$$\begin{aligned} A^{-1/2}(B_{X}^{n}(\sqrt{\hbar }))\times A^{1/2}(B_{P}^{n}(\sqrt{\hbar }))=M_{A^{1/2}}(B_{X}^{n}(\sqrt{\hbar })\times B_{P}^{n}(\sqrt{\hbar })) \end{aligned}$$

where $$M_{A^{1/2}}= \begin{pmatrix} A^{1/2} &{}\quad 0\\ 0 &{}\quad A^{-1/2} \end{pmatrix}$$ is a symplectic dilation. Using again (36) the John ellipsoid of this state is

$$\begin{aligned} (X\times X^{\hbar })_{\text{John}}=M_{A^{1/2}}(B^{2n}(\sqrt{\hbar })) \end{aligned}$$

and to the latter corresponds the function with Wigner transform

$$\begin{aligned} W\psi (z)=(\pi \hbar )^{-n}\exp -\left[ \frac{1}{\hbar }(Ax\cdot x+A^{-1}p\cdot p)\right] \end{aligned}$$

and hence, up to an irrelevant constant phase $e^{i\gamma }$,

$$\begin{aligned} \psi (x)=\psi _{A,0}(x)=\left( \tfrac{1}{\pi \hbar }\right) ^{n/4}(\det A)^{1/4}e^{-\tfrac{1}{2\hbar }Ax\cdot x}. \end{aligned}$$

These examples suggest that there is a deeper underlying structure relating elliptic quantum states to Gaussian wavefunctions. To study this relation we begin by defining an equivalence relation on ${\text {Quant}}_{0}(n)$: We will say that two states $X_{\ell _{1}}\times X_{\ell _{1}^{\prime }}^{\hbar }$ and $X_{\ell _{2}}\times X_{\ell _{2}^{\prime }}^{\hbar }$ are unitarily equivalent and write

$$\begin{aligned} X_{\ell _{1}}\times X_{\ell _{1}^{\prime }}^{\hbar }\overset{U(n)}{\sim } X_{\ell _{2}}\times X_{\ell _{2}^{\prime }}^{\hbar } \end{aligned}$$

if there exists a symplectic rotation $U\in U(n)$ such that $(\ell _{1},\ell _{1}^{\prime })=U(\ell _{2},\ell _{2}^{\prime })$ and

$$\begin{aligned} X_{\ell _{1}}\times X_{\ell _{1}^{\prime }}^{\hbar }=U(X_{\ell _{2}}\times X_{\ell _{2}^{\prime }}^{\hbar }). \end{aligned}$$

Since *U*(*n*) is a group the relation $\overset{U(n)}{\sim }$ enjoys the properties of reflexivity, symmetry, and transitivity, and is thus indeed an equivalence relation. We denote by $\widetilde{X_{\ell }\times X_{\ell ^{\prime }}^{\hbar }}$ the equivalence class of the state $X_{\ell }\times X_{\ell ^{\prime }}^{\hbar }$ for this relation and by ${\text {Quant}}_{0}(n)/U(n)$ the set of all such equivalence classes. Recall (formula 35) that we have identified ${\text {Quant}}_{0}(n)$ with ${\text {Sp}}(n)/O(n)$. Following result identifies ${\text {Gauss}}_{0}(n)$ with ${\text {Quant}}_{0}(n)/U(n)$:

#### Proposition 18

There is a canonical identification

50 $$\begin{aligned} {\text {Gauss}}_{0}(n)\equiv {\text {Quant}}_{0}(n)/U(n) \end{aligned}$$

between the set of centered Gaussian wavepackets $\psi _{AB}$ and the equivalence classes $\widetilde{X_{\ell }\times X_{\ell ^{\prime }}^{\hbar }}$ of centered elliptic quantum states. More generally we have an identification

51 $$\begin{aligned} {\text {Gauss}}(n)\equiv {\text {Quant}}(n)/U(n) \end{aligned}$$

#### Proof

Let $\psi _{A,B}\in {\text {Gauss}}_{0}(n)$ be a Gaussian wavepacket and

$$\begin{aligned} W\psi _{AB}(z)=(\pi \hbar )^{-n}e^{-\frac{1}{\hbar }Gz\cdot z}, G=(SS^{T})^{-1} \end{aligned}$$

its Wigner transform. The ellipsoid $\{z:Gz\cdot z\le \hbar \}$ is the quantum blob $Q=S(B^{2n}(\sqrt{\hbar }))$, and in view of Proposition 16 the latter is the John ellipsoid of the state

$$\begin{aligned} X_{\ell }\times X_{\ell ^{\prime }}^{\hbar }=S(B_{X}^{n}(\sqrt{\hbar })\times B_{P}^{n}(\sqrt{\hbar })), \\ \ell =S\ell _{X}, \ell ^{\prime }=S\ell _{P}. \end{aligned}$$

If $S^{\prime }\in {\text {Sp}}(n)$ is another symplectic matrix such that $G=(S^{\prime }(S^{\prime })^{T})^{-1}$ then $S^{\prime }=SU$ for some symplectic rotation $U\in U(n)$ and hence $S^{\prime }(B^{2n}(\sqrt{\hbar }))=S(B^{2n} (\sqrt{\hbar }))$ so that *Q* is also the John ellipsoid of the state

$$\begin{aligned} X_{\ell _{1}}\times X_{\ell _{1}^{\prime }}^{\hbar }=S^{\prime }(B_{X}^{n} (\sqrt{\hbar })\times B_{P}^{n}(\sqrt{\hbar })),\\ \ell _{1}=SU\ell _{X}, \ell _{1}^{\prime }=SU\ell _{P}. \end{aligned}$$

Conversely, let $X_{\ell }\times X_{\ell ^{\prime }}^{\hbar }$ be a centered elliptic quantum state and choose $S\in {\text {Sp}}(n)$ such that $(\ell ,\ell ^{\prime })=S(\ell _{X},\ell _{P})$ and

52 $$\begin{aligned} X_{\ell }\times X_{\ell ^{\prime }}^{\hbar }=S(B_{X}^{n}(\sqrt{\hbar })\times B_{P}^{n}(\sqrt{\hbar })) \end{aligned}$$

(Proposition 13). In view of Proposition 16 the John ellipsoid of $X_{\ell }\times X_{\ell ^{\prime }}^{\hbar }$ is the quantum blob $Q=S(B^{2n}(\sqrt{\hbar }))$, hence to $X_{\ell }\times X_{\ell ^{\prime }} ^{\hbar }$ corresponds the generalized Gaussian $\psi _{AB}$ with Wigner transform

$$\begin{aligned} W\psi _{AB}(z)=(\pi \hbar )^{-n}e^{-\frac{1}{\hbar }Gz\cdot z}, G=(SS^{T})^{-1}. \end{aligned}$$

We may replace $X_{\ell }\times X_{\ell ^{\prime }}^{\hbar }$ with

$$\begin{aligned} X_{\ell _{1}}\times X_{\ell _{1}^{\prime }}^{\hbar }=S^{\prime }U(B_{X}^{n} (\sqrt{\hbar })\times B_{P}^{n}(\sqrt{\hbar })),\ell _{1}=SU\ell _{X}, \ell _{1}^{\prime }=SU\ell _{P}. \end{aligned}$$

with $U\in U(n)$ without altering *G*, hence $W\psi _{AB}$ (and thus $\psi _{AB}$) only depends on the equivalence class $\widetilde{X_{\ell }\times X_{\ell ^{\prime }}^{\hbar }}$. The extension of (50) to formula (51) is straightforward. $\square$

## Perspectives for a Generalization

So far we have been considering Lagrangian products of ellipsoids. The next— and fundamental!—step would be to generalize our constructions to products $X\times X^{\hbar }$ (or, more generally, $X_{\ell }\times X_{\ell ^{\prime } }^{\hbar }$) where *X* or $X_{\ell }$ is not an ellipsoid, but an arbitrary convex set, leading to a non-Gaussian quantum state. It is clear why this problem has such an overwhelming importance since it opens the door to a general geometric theory of quantum states. This problem will be addressed in forthcoming work: let us just outline here some of the difficulties inherent to such an extension of our theory. So, we would like to construct a generalization of ${\text {Quant}}(n)$ where the Lagrangian quantum states are represented by arbitrary convex sets. The first mathematical difficulty that occurs is the determination of the point with respect to which the polar dual should be calculated. Let in fact $X(x_{0})$ be an arbitrary convex body in $\ell _{X}={\mathbb {R}}_{x}^{n}$; by definition its centroid (or barycenter) is

53 $$\begin{aligned} x_{0}=\frac{1}{{\text {Vol}}_{n}(X)}\int _{X}x_{1}dx_{1} +\cdots +x_{n}dx_{n}=0. \end{aligned}$$

It is easily verified that if *X* is an ellipsoid, then the centroid coincides with its center in the usual sense. To define the polar dual of $X(x_{0})$ one is tempted to use the same procedure as for ellipsoids and to define $X(x_{0})^{\hbar }$ as the dual of the centered convex body $X=T(-x_{0} )X(x_{0})$. However this is not the good choice. Here is why: when we defined the polar of an ellipsoid by using a translation to make it centered at the origin it turns out that the Blaschke–Santaló product ${\text {Vol}}_{n}(X(x_{0})){\text {Vol}}_{n} (X^{\hbar }(x_{0}))$ attains the value $({\text {Vol}}_{n} B^{n}(\sqrt{\hbar })^{2}$. The difficulty comes from the fact that in the general case of arbitrary convex body we need to choose the correct center with respect to which the polarity is defined since there is no privileged “center”; different choices may lead to polar duals with very different volumes (see Example 26). Santaló proved in [24] the following remarkable result: there exists a *unique* interior point $x_{\text{S}}$ of *X* (the “Santaló point of *X*”) such that the polar dual $X^{\hbar }(x_{\text{S}})=(X-x_{\text{S}})^{\hbar }$ has centroid $\overline{p}=0$ and its volume ${\text {Vol}}_{n}(X^{\hbar }(x_{\text{S}}))$ is *minimal* for all possible interior points $x_{0}$:

54 $$\begin{aligned} {\text {Vol}}_{n}(X){\text {Vol}}_{n}(X^{\hbar }(x_{\text{S}}))\le ({\text {Vol}}_{n}B^{n}(\sqrt{\hbar } )^{2} \end{aligned}$$

with equality if and only if *X* is an ellipsoid. We note that the practical determination of the Santaló point is in general difficult and one has to use ad hoc methods in each particular case. See [2] for a discussion of this issue.

Having in mind that the polar dual is calculated with respect to the Santaló point (not the centroid!) we can define the associated canonical Lagrangian quantum state exactly as follows let $(\ell _{X},\ell _{P} )\in {\text {Lag}}_{0}^{2}(n)$, *be* the canonical Lagrangian frame and $X(x_{\text{S}})\in {\text {Conv}}(\ell )$ a convex body carried by $\ell _{X}$ and with Santaló point $x_{\text{S}}$. The associated Lagrangian state is then

$$\begin{aligned} X(x_{\text{S}})\times (X(x_{\text{S}})-x_{\text{S}})^{\hbar } +p_{0})=X(x_{\text{S}})\times X^{\hbar }(p_{0}) \end{aligned}$$

and we again have a fiber bundle structure

$$\begin{aligned} \pi :{\text {Quant}}_{0}(n)\longrightarrow {\text {Conv}} (\ell ). \end{aligned}$$

The study of the latter is less straightforward than in the case of ellipsoids, and will be done in a forthcoming work. We also notice that we can associate to every state an ellipsoid using the John ellipsoid method, but the role played by the latter is unclear (it is not quite obvious that it should be a quantum blob; if it were the case it could correspond to the covariance matrix of the state). At this point one might want to use the theory of the Minkowski functional to give a geometric study; this leads us to consider difficult non-linear problems. All this is also related to the powerful notion of symplectic capacity, which we discussed in [16] following the ideas in [2–4] All these questions are fascinating ad answers might lead to a geometric reformulation of quantum mechanics where the notion of polar duality in a sense replaces the usual uncertainty principle. We will come back with answers in a near future.
